# A Case of Pure Pericarditis Associated With COVID-19: Application of Classical Clinical Evaluation for Differential Diagnosis

**DOI:** 10.7759/cureus.37794

**Published:** 2023-04-18

**Authors:** George W Pettit, Leisa M Eubank, Joshua Richard, Ethan T Brown, Rangaraj R Gopikishan

**Affiliations:** 1 Emergency Department, ERKaty, Katy, USA; 2 Family Medicine, Right Choice Urgent Care, Cypress, USA; 3 Medicine, Houston Methodist Willowbrook Hospital, Houston, USA

**Keywords:** ekg abnormalities, st-elevation myocardial infarction (stemi), sars-cov-2, covid-19, pericardial diseases

## Abstract

Pericarditis of varying severity is being recognized as a rare complication of the COVID-19 infection. We present a patient with an electrocardiogram (EKG) and physical exam findings that initially seemed to most likely be pericarditis related to the COVID-19 infection. The differential diagnosis was a bit difficult because it included ST-segment elevation myocardial infarction (STEMI) due to some EKG changes and early repolarization changes that were rather robust. Treatment options for STEMI could cause severe harm if the process turned out to be pericarditis. Treatment options for pericarditis could cause severe harm if the process turned out to be STEMI. And treatment options for early repolarization might be no treatment at all, which could cause harm if the process turned out to be STEMI or pericarditis. In this case, a correct diagnosis was very important to ensure a good clinical outcome. We would like to share our thought processes in the management of this case.

## Introduction

While the COVID-19 pandemic has been ongoing for over two years, there is emerging evidence to suggest that pure acute pericarditis, without preceding myocarditis, may be a more frequent extrapulmonary manifestation of SARS-CoV-2 infection. In fact, in a meta-analysis of chest computed tomography (CT) scans that included 2738 patients with COVID-19 infections, 4.55% had pericardial effusions [1]. It is important to note, however, that pericardial effusion does not necessarily mean pericarditis is present. In fact, in a study of 173 patients with large pericardial effusions, only 12% had acute pericarditis [2]. In another study, which involved 139 healthcare workers with past confirmed COVID-19 infection (9.3-11 weeks earlier), 14.5% had evidence of prior pericarditis or myopericarditis when assessed with classical criteria or cardiac magnetic resonance imaging [3]. Furthermore, in a study that recruited 48 student-athletes recovering from COVID-19 infection who returned to their university campus in July 2020, it was found that 19 out of 48 students (39.6%) had an echocardiogram and/or sequential cardiac magnetic imaging studies that showed imaging features of resolving pericardial inflammation [4]. We were inspired by the case report published in 2020 by Fox et al. [5], in which they described in much excellent detail the clinical course of a young, healthy man with acute effusive pure pericarditis with pericardial tamponade, most likely a result of a preceding COVID-19 infection.

We present the case of a healthy 36-year-old man with few comorbidities, a body mass index (BMI) of 32, and mild hypertension who presented to the emergency room (ER) with chest pain and whose electrocardiogram (EKG) showed early repolarization changes plus features consistent with pericarditis or acute ST-elevation myocardial infarction (STEMI). For us, differentiation of these disparate findings was very important since treatment for pericarditis with colchicine and non-steroidal anti-inflammatory drugs (NSAIDs) could cause harm if the process turned out to be STEMI; in that case, colchicine would not help, revascularization treatment would be delayed, and NSAIDs could cause further harm if given to a patient with STEMI [6]. Conversely, treatment of STEMI (with anticoagulants and nitroglycerine) could cause harm if the process turned out to be pericarditis; in that case, treatment with colchicine and/or NSAIDs would be delayed, anticoagulants might predispose to a hemorrhagic pericardial effusion, and nitroglycerine would further worsen tamponade physiology by reducing preload. Finally, if the patient’s EKG findings were decided to be due to early repolarization, we might disregard the consideration that they were possibly due to STEMI or pericarditis.

With the use of serial EKGs obtained during our patient’s illness and after complete symptomatic recovery, we attempted to use some classical and time-honored EKG features to sort out these findings. In a patient with chest pain plus early repolarization plus EKG findings consistent with pericarditis or STEMI, we found this to be challenging. Many more cases of pure pericarditis are likely to occur with COVID-19 infections.

## Case presentation

A 36-year-old man presented to an ER with an eight-hour history of intermittent "fluttering and stabbing" chest pain with radiation to the left shoulder. He described the pain as "much worse" in the supine position and "better" when sitting up. He denied any recent fevers or respiratory symptoms. He did admit that he had tested positive for SARS-CoV-2 four weeks earlier; he noted that at that time he was symptom-free. He underwent quarantine at home for four weeks and never developed any severe symptoms, except he noted that he felt "tired" since the COVID-19 infection. He denied known sick contacts or recent travel. His only medicines were for hypertension and were amlodipine 5 mg daily and carvedilol 25 mg every 12 hours. He admitted to being often non-adherent with those medicines, and he had not taken any medications for his blood pressure recently. The patient noted a history of allergy to lisinopril and ibuprofen; he was not sure about allergies to other NSAIDs. He stated that he took 162 mg of aspirin before coming to the ER and had no reaction to that. He denied any history of autoimmune problems or known exposure to other infectious diseases. He denied any history of alcohol, tobacco, or drug abuse. The patient noted that his father had a myocardial infarction and required stents when he was in his 30s.

On arrival at the free-standing ER, the patient’s blood pressure was 162/96 mmHg, heart rate was 90 bpm, respiratory rate was 20 rpm, the oral temperature was 98.3 °F, pulse oximetry on room air was 97%, weight was 192 lbs, and height was 5 feet 6 inches (BMI was 31.9). On the physical exam, he was awake, alert, and oriented to person, place, and time. His self-assessed pain level was 8 out of 10. The head-eyes-ears-nose-throat (HEENT) exam was unremarkable. The neck was supple and without evidence of jugular venous distention (JVD); no carotid bruits were present. He was not in respiratory distress. The chest was non-tender, and the breath sounds were normal. On the initial cardiac exam, there was no evidence of cardiomegaly; the point of maximal impulse was non-displaced. There were no murmurs or gallops. Auscultation of the heart in supine, sitting up, and leaning forward while sitting up positions did not reveal a pericardial friction rub. Bowel sounds were normal; there were no vascular bruits, and there was no abdominal tenderness. The extremities showed no edema, both legs were not tender, and Homan’s sign was negative. The neurological exam was normal. On a repeat cardiac exam two hours after arrival, a three-component pericardial friction rub was auscultated when the patient was sitting up and leaning markedly forward.

**Figure 1:**
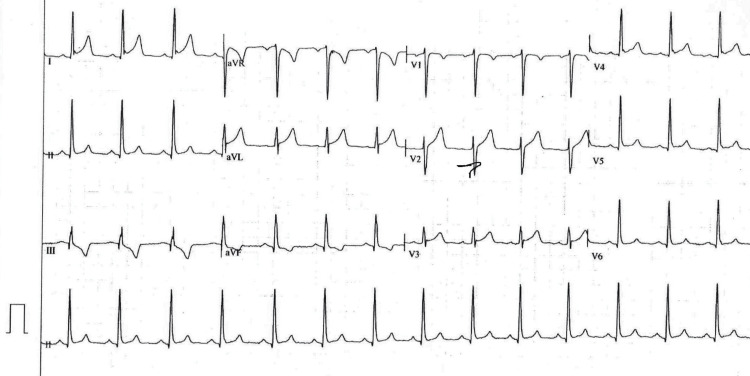
Initial 12 lead electrocardiogram. Initial 12 lead EKG shows sinus rhythm with rates in the 80s. There was diffuse ST-segment elevation that was concave upward in leads I, II, aVL, and V2 through V5, and all ST segment elevation was less than 5 mm. There was ST-segment depression in AVR. There was ST-segment depression in lead III and ST elevation in its reciprocal lead, AVL. ST-segment elevation was greater in II than in III (ST segment was actually depressed in III). There was no definite significant variance from isoelectric in the PR segment in any lead.

**Figure 2:**
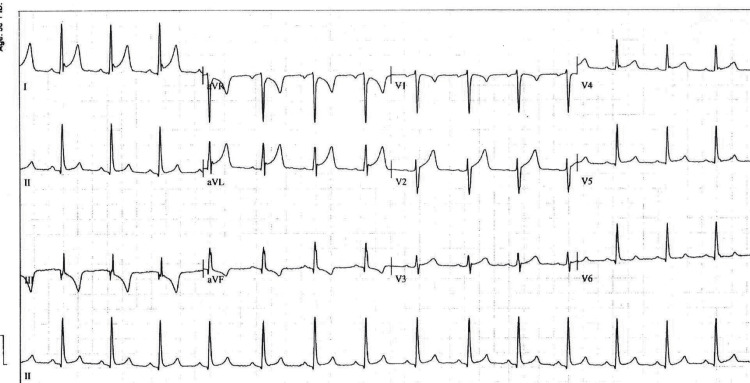
12 lead electrocardiogram taken three hours after initial electrocardiogram. The EKG changes possibly consistent with STEMI or pericarditis seemed to be generally more intense, when compared to the EKG changes in Figure 1 (the patient's initial EKG). We discussed this in greater detail in our "Discussion" section.

The initial 12-lead EKG (Figure 1) and an EKG three hours later (Figure 2) showed sinus rhythm with rates in the 80s. The TP-segment was assumed to be isoelectric for all measurements, and ST-segment changes were measured at the "J point." There was diffuse ST segment elevation that was concave upward in leads I, II, AVL, and V2 through V5, and all ST-segment elevation was less than 5 mm. There was ST-segment depression in AVR. There was ST segment depression in lead III and ST elevation in its reciprocal lead, AVL. ST-segment elevation was greater in II than in III (the ST segment was actually depressed in III). There was no definite, significant variance from isoelectric in the PR segment in any lead (Figures 1-2).

A complete blood count showed a normal white blood cell count (6680/µL) with a normal differential, including a lymphocyte count of 3040/µL. Hemoglobin and hematocrit were normal. Serum chemistries were normal. Troponin I levels done upon admission to the ER and then 3, 12, and 21 hours later were all non-detectable. D-dimer and BNP (brain natriuretic peptide) were both measured at zero upon arrival at the ER. Coronavirus NAA (a multitarget PCR test developed by Lab Corp that provides definitive reporting for the detection of SARS-CoV-2) was positive. The chest radiograph was normal. A transthoracic echocardiogram showed normal left ventricular systolic function with an ejection fraction of 55% to 60%. There was no pericardial effusion. The chest CT scan with contrast showed normal pulmonary arteries and no pulmonary emboli. The heart appeared normal, with no cardiomegaly and no pericardial effusion.

**Figure 3:**
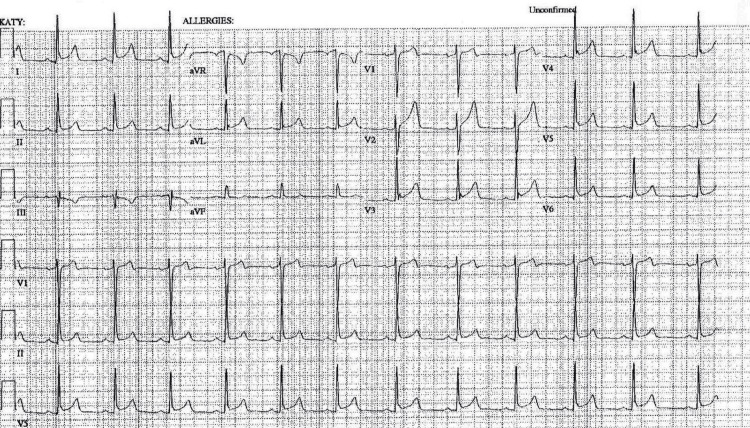
Electrocardiogram taken 15 hours after the patient's initial electrocardiogram in the emergency room (at a time when his symptoms were noticeably improved). This 15-hour EKG shows some resolution of changes consistent with pericarditis and no progression of the reciprocal changes which might have reflected a high lateral STEMI (ST elevation an AVL, and ST depression in lead III). This is discussed in more detail in our "Discussion" section.

The patient was admitted to the hospital and started on oral colchicine, 0.6 mg every 12 hours; NSAIDs were withheld because of his allergy (with probable anaphylaxis in the past) to ibuprofen. While in the hospital and taking colchicine, the patient had rapid improvement in his symptoms. The three-component pericardial rub that was heard in the ER was no longer present. An EKG taken later (15 hours after his first EKG in the ER), when his symptoms were noticeably improved, showed some resolution of changes consistent with pericarditis and no progression of the reciprocal changes, which, if present, may have reflected a high-lateral STEMI (ST elevation in the AVL and depression in lead III; Figure 3).

**Figure 4:**
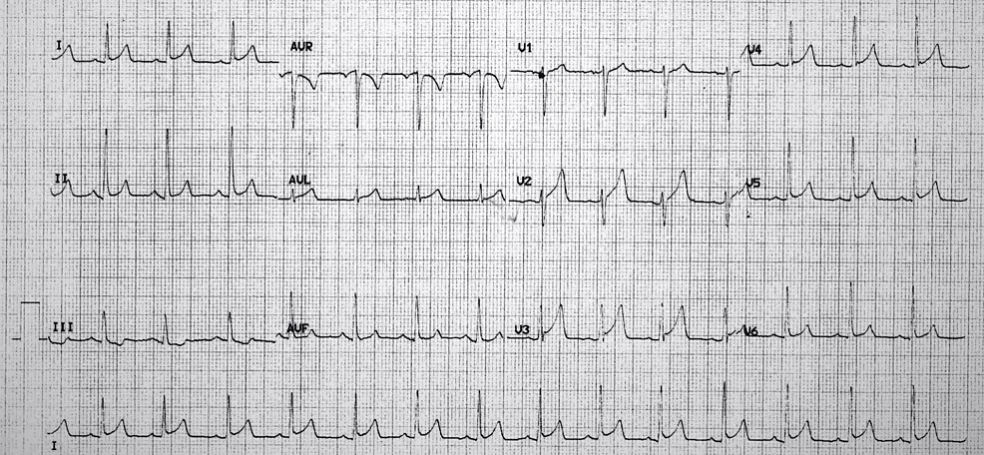
This electrocardiogram was obtained at seven weeks after his hospital admission. He was symptom free at that time. This subsequent EKG showed normalization of the ST depression in lead III that made "high lateral STEMI" a concern. EKG changes consistent with pericarditis were gone, and the EKG interpretation was "early repolarization." In the Discussion section, we discussed this in more detail.

The patient was discharged home, where he made a full recovery over the next two weeks. A subsequent EKG obtained as an outpatient seven weeks after his admission showed normalization of the ST depression in lead III that had made "high lateral STEMI" a concern. The aforementioned EKG changes consistent with pericarditis were gone, and the EKG interpretation was "early repolarization" (Figure 4).

## Discussion

In pericarditis, the chest pain tends to be pleuritic and sharp, and it typically worsens in the supine position and is lessened by sitting up and leaning forward. These two features were present in our patient. Also, our patient had an evanescent three-component pericardial friction rub early in the course of his disease; that rub is highly specific for acute pericarditis. Our patient had a normal echocardiogram without evident pericardial effusion, but that is often the case (about 40%) in patients with pericarditis. Likewise, a CT scan with IV contrast showed no evidence of pericarditis (pericardial effusion or pericardial enhancement); however, the sensitivity of a CT scan with IV contrast (PE protocol) for detection of pericarditis is only about 55%.

As disease processes, STEMI, pericarditis, and early repolarization are sometimes difficult to differentiate. All three can result in ST-segment elevation, but the characteristics of and reasons for the elevation differ. STEMI causes ST elevation as a consequence of subendocardial injury. Pericarditis causes ST elevation as a consequence of epicardial inflammation. Therefore, these are two totally different processes that result in similar, but not identical, ST segment changes. Early repolarization causes ST segment changes also, but may not be a disease process for most patients. In order to differentiate STEMI from pericarditis, we have to rely on some rather subtle differences in the EKG. ST-segment elevation or depression is determined relative to the T-P segment, which is considered to be isoelectric. The ST segment level is considered the J-point. Regarding the ST segment, an elevation of more than 5 mm makes pericarditis less likely and STEMI more likely. ST segments tend to be concave upward with pericarditis and convex (tombstone) upward with STEMI. With STEMI, we can often use ST elevation and reciprocal changes (ST segment depression) in opposite leads to localize the injury to a specific coronary artery distribution. In pericarditis, the ST elevation tends to be diffuse (except in AVR, where the ST segment is usually depressed). A differentiation between ventricular cavity leads (AVR and sometimes V1 and V2) and epicardial leads (the remainder of leads in the frontal and horizontal planes) is helpful for understanding the ST segment changes that occur in pericarditis {8}.

The PR depression in epicardial leads that often accompanies early pericarditis is a consequence of atrial epicardial inflammation, which manifests as PR depression in epicardial leads and may manifest as PR elevation in the cavity leads (AVR and V1). For anatomical reasons related to the positions of the atrial chambers, this is in contrast to ventricular epicardial inflammation, which manifests as ST-segment elevation in the epicardial leads and oftentimes ST depression in the cavity leads. In fact, ST-segment elevation in AVR can exclude pericarditis and make STEMI more likely. Leads V1 and V2 can be cavity leads like AVR, and they can exhibit these same findings. In short, in pericarditis, the ST and PR segment changes are all due to pericardial inflammation, but due to differences in the anatomical positioning of the atrial and ventricular chambers, the segment changes in the atria (PR segment) and ventricles (ST segment) are in opposite directions.

In the case of our patient, the initial EKGs (Figures 1-2; two hours later) showed changes consistent with elements of early repolarization, pericarditis, and STEMI. Changes consistent with early repolarization were ST-segment elevation (less than 3 mm) in leads V1 through V4, which were concave upward and most marked in V2, plus a notch at the J-point of V4.

Findings consistent with pericarditis (Figures 1-2) were present as follows. The ST segment elevations were diffuse in epicardial leads; they were all less than 5 mm, and all ST segment elevations were convex upward. The ST segment was depressed in AVR, consistent with pericarditis. Rather than the PR interval depressions that are often seen with early pericarditis, the PR segments in our patient were consistently isoelectric in all leads in all of the EKGs done; in Spodick’s seminal study (published in 1973) on electrocardiographic sequences in pericarditis, he found that also to be the case in 9 of the 50 pericarditis cases that he studied [7,8]. Furthermore, the ST segment depression and T wave inversion in III plus the ST elevation in AVL, which gave us concern for injury in AVL with reciprocal change in III, were noted by Spodick [8] in 7 of his 50 pericarditis patients, and the axis of the ST segment for those patients was always between 0 degrees and +20 degrees in the frontal plane. For our patient, that ST segment axis was 0 degrees. Thus, we were a bit relieved that the ST depression that we found in III was also noted by Dr. Spodick in 14% of his pericarditis patients.

Findings concerning STEMI included progression (over three hours, from Figures 1-2) of ST-segment elevation in lead 1 and AVL plus a markedly deeper T wave in III in the three-hour later EKG (Figure 2). In fact, these changes prompted the computer in our EKG machine to change its diagnosis to "acute MI" and "lateral injury." These changes all became much less marked in an EKG 12 hours later (Figure 3) and were totally resolved in a convalescent EKG seven weeks later (Figure 4). These serial EKGs, along with a normal transthoracic echocardiogram with normal systolic function and undetectable troponin I and multiple serial measurements, helped assure that this patient did not have a STEMI.

By using the patient’s convalescent EKG (Figure 4) and comparing it to EKGs obtained during his illness (Figures 1-3), we were able to retrospectively separate EKG changes of pericarditis from the patient’s early repolarization changes. In a study in 1982 [9], Ginzton and Laks were able to perfect a tool that differentiated pericarditis from early repolarization by employing some simple measurements made on EKGs. They showed that the ratio of the heights of the ST and T waves from various EKG leads could be used to do this. In fact, if that ratio was greater than or equal to 0.25 in V6, the positive predictive value (PPV) and negative predictive value (NPV) favored pericarditis, each with a value of 1. If values were less than 0.25, that would favor the diagnosis of early repolarization. If accurate measurements for V6 were not obtainable (as is often the case), then V4, V5, and lead I could be used, but their PPV and NPV values would be slightly less than 1. For our patient, during the early hours of his illness (Figures 1-2), all values for ST/T were greater than or equal to 0.25 (Tables 1-2); in V6, the values for both EKGs were 0.25, but the measured values were too small, and those values for V6 should be considered likely inaccurate. So that would favor pericarditis rather than early repolarization early in his illness.

**Table 1:** Data from electrocardiogram in Figure 1.

	Figure 1 (7/12/2020 at 21:20) (active illness)		
Lead	ST	T	ST/T
I	2	7	0.29
V4	1.5	4	0.38
V5	1	2.5	0.4
V6	0.5	2	0.25

**Table 2:** Data from the electrocardiogram in Figure 2.

	Figure 2 (7/13/2020 at 00:18) (active illness)		
Lead	ST	T	ST/T
I	2.5	9	0.28
V4	1.5	3	0.5
V5	1	2	0.5
V6	0.25	1	0.25

In the last EKG, which we obtained during his active illness (Figure 3 and Table 3), ST/T in lead I and V6 were both less than 0.25, but in V4 and V5, those values were equal to 0.25. These reductions in those ST/T values correlated with his reduction in symptoms from his illness at that time. On his convalescent EKG (Figure 4), all values were less than 0.25 (Table 4), so that would strongly favor early repolarization (only) after his recovery.

**Table 3:** Data from the electrocardiogram in Figure 3.

	Figure 3 (7/13/2020 at 11:45) (active illness)		
Lead	ST	T	ST/T
1	1	5	0.2
V4	1	4	0.25
V5	1	4	0.25
V6	0.5	3	0.17

**Table 4:** Data from the electrocardiogram in Figure 4.

	Figure 4 (9/5/20:20)		
Lead	ST	T	ST/T
I	1	4.5	0.22
V4	1	6	0.17
V5	1	4.5	0.22
V6	0.5	3	0.17

Thus, we feel confident that the EKG changes that we observed during the acute phase of this patient’s illness were due to pericarditis in addition to his baseline early repolarization, which, of course, persisted six weeks later when he was completely recovered and asymptomatic.

Recent evidence and thought challenge the notion that early repolarization is always a benign process. So we elected to describe our patient’s baseline EKG findings as "early repolarization" and not "normal variant." Investigators have identified subsets of patients with early repolarization who may be prone to ventricular fibrillation and sudden death. At this point in time, much of the ongoing research in this area is aimed at the risk stratification of patients with early repolarization. This research is progressing along several lines [10,11]. Work done in 2011 by Tikkanen et al. [12] and in 2016 by Viskin et al. [13] has indicated that an upward-sloping ST segment followed by an upright T-wave in the presence of end QRS notching is benign (as we see in our patient's convalescent EKG, V4 in Figure 4). However, early repolarization with a horizontal or downward-sloping ST segment is potentially more serious and more likely to be associated with sudden death. As this area of research progresses, it will be exciting to watch a risk stratification process develop along with action plans that we can implement to protect our patients if their risk is high.

The possibility that our patient’s pericarditis was caused by SARS-CoV-2 is complex to sort out. He had tested positive for that virus four weeks prior to his presentation to us, but other than persistent "tiredness," he had no other symptoms since then. He tested positive for SARS-CoV-2 (PCR test) on admission to our hospital, but it is difficult to know if he had become reinfected or had an unusually persistent case that started from his infection four weeks prior. Diaz-Arocutipa et al. [14] surmised that in COVID-19 patients with pericarditis, there are two possible scenarios to consider. The first is the patient being treated for pericarditis who subsequently becomes infected with COVID-19. The second is the patient with COVID-19 who develops pericarditis. Perhaps our patient would most likely fall into the second category. If that were the case, a persistent COVID-19 infection would most likely be the cause of the pericarditis. So, in this case, the pericarditis would have been persistent for four weeks, which seems a bit unusual. Germane to this is the work done in a subsequent large study (734 articles reviewed), which led to 33 case reports (a total of 34 patients) with myopericarditis or pericarditis identified [14]. Of those patients, ten (30%) were found to have pure pericarditis diagnosed after COVID-19 infection, with a range of 5-56 days after an initial positive COVID-19 test. Since our patient presented with pericarditis one month after a positive COVID-19 test, he would fall within that 5-56-day range. Our patient had no pericardial effusion (by transthoracic echo or CT scan), so pericardiocentesis was not considered, and isolation of COVID-19 from pericardial fluid was not done. The difficulties encountered in isolating SARS-CoV-2 from the pericardial fluid are myriad and well described in work by Fox et al. [5]. It is difficult to isolate or detect SARS-CoV-2 in pericardial fluid, so it is difficult to prove that an effusion is due to that virus. Pertinent to this discussion is the work by Kaminski et al. [15]. This is a case report that describes an interesting case of pericarditis with positive COVID testing (by nasopharyngeal swab) that occurred about 10 weeks after recovery from a very mild COVID infection. The authors of that case report bring up the possibility that his second COVID infection might have been either recurrent or atypical latent infection. This idea expands on the aforementioned thoughts of Diaz-Arocutipa et al. by allowing for either a recurrent or atypical latent infection that preceded the pericarditis. Our patient was similar to the patient of Kaminski et al., in that he had a nearly asymptomatic case of COVID before he presented to us with positive COVID testing and pericarditis, except their patient was 65 years old and also had multiple other comorbidities.

An excellent case report in 2022 by Amoateng et al. [16] detailed the evaluation of a 19-year-old man with chest pain who had recovered from COVID pneumonia just six weeks prior to his presentation. The patient presented with some clinical features consistent with pericarditis and an EKG with changes suspicious for pericarditis but more consistent with early repolarization. Apparently, this evaluation and workup took place in a very medically underserved community. Despite that disadvantage, the clinicians made excellent use of the resources that they had. They were able to render impressive care by utilizing clinical skills plus the use of basic resources such as serial EKGs, identifying very subtle EKG changes, using point-of-care ultrasound, and applying the Ginzton Electrocardiographic Criteria to rule in or rule out pericarditis versus early repolarization.

## Conclusions

A small but significant number of patients with COVID-19 infection have pericardial involvement as part of their illness. Our young, healthy patient with pericarditis also had EKG findings of early repolarization and, more concerning, had EKG findings that could have been STEMI. We point out that promptly arriving at the correct diagnosis in this situation is very important before treatment is initiated. From several aspects, our patient’s presentation was a bit unusual. Our case report highlights the importance of promptly arriving at a correct diagnosis before initiating treatment, and we hope our description of this case can be helpful to others.
